# Stress adaptation is associated with insulin resistance in women with gestational diabetes mellitus

**DOI:** 10.1038/s41387-020-0107-8

**Published:** 2020-01-22

**Authors:** Yan Feng, Qi Feng, Hongmei Qu, Xinna Song, Jianwei Hu, Xiaomeng Xu, Li Zhang, Shaohua Yin

**Affiliations:** 1 Department of Clinical Nutrition, Yuhuangding Hospital affiliated to Qingdao University, #20 East Yuhuangding Road, 264000 Yantai, China; 2 Department of General Surgery, CPLA No. 71897, #1 Bayi Road, 710000 Xi’an, China; 3 Kunshan Maternal and Child Health Hospital, #458 Tongfeng Road, 215301 Kunshan, China; 4 Jining First People’s Hospital, #6 Jiankang Road, 272011 Jining, China; 5 Department of Outpatient, Yuhuangding Hospital affiliated to Qingdao University, Yantai, China

**Keywords:** Endocrinology, Medical research

## Abstract

**Aim:**

Oxidative stress is known to increase the risk of insulin resistance (IR). The aim of this study was to investigate the association between stress hormones and IR in women with gestational diabetes mellitus (GDM), in an attempt to gain insights into the pathogenesis of GDM.

**Methods:**

Recruited in this study were 70 GDM women and 70 healthy pregnant women as control. Malondialdehyde (MDA), superoxide dismutase (SOD), glutathione (GSH), plasma epinephrine (E), noradrenaline (NE), glucagon, and cortisol levels were detected. IR was assessed by homeostasis model assessment of IR (HOMA-IR) in both groups. Correlations among stress hormones, oxidative stress, and IR were analyzed by Pearson’s correlation after log transformation.

**Results:**

Compared with the Control group, MDA was increased and anti-oxidative enzymes SOD and GSH were decreased significantly in the GDM group. Glucagon, E, and NE in the GDM group were increased by 22.42%, 36.82%, and 35.09%, respectively, as compared with those in the Control group. MDA showed a significant positive correlation, and SOD showed a negative correlation with HOMA-IR in the GDM group. In addition, HOMA-IR was positively related to glucagon, E, NE, and cortisol.

**Conclusions:**

Elevation of stress hormones and stress adaptation disturbance may be associated with the pathogenesis of GDM in pregnant women.

## Introduction

Gestational diabetes mellitus (GDM) is characterized by abnormal glucose tolerance during pregnancy. The prevalence of GDM is increasing worldwide, and about 2–14% pregnancies were affected by GDM depending on different criteria, not only including detection types and diagnostic glucose threshold levels but also because of screening policies, screening routes, and the prevalence of type 2 diabetes in populations^1^. GDM increases the risk of occurrence of type 2 diabetes mellitus in both mothers and their offspring. The etiology of GDM remains unclear^2^.

There exists a progressive decrease in insulin sensitivity throughout pregnancy, and it is evident that insulin resistance (IR) is associated with GDM because of increased maternal adiposity and the hormonal effects of the placenta; glucose clamps in pregnant women have documented in a very convincing way changes in IR during physiological pregnancy and pregnancy with GDM^3^. Several studies have demonstrated that oxidative stress (OS) is one of the most important mechanisms underlying IR^4–6^. Some other studies have also shown that OS increased the risk of new-onset DM 2, leading to hyperglycemia^7,8^. We also examined OS injury indexes in GDM patients and analyzed the relationship between these indexes and IR.

In addition, pregnant women are usually in a stressful condition, which together with increased requirements on oxygen and energy during pregnancy may lead to increased OS injury^9^. Is it possible that changes in stress hormones during pregnancy may also contribute to IR other than OS? Our previous study suggested that stress adaptation disturbance during pregnancy may aggravate stress-induced hyperglycemia in rats^10,11^. The aim of the present study was to document the association between stress hormones and IR in women with GDM, in an attempt to gain insights into the pathogenesis of GDM.

## Research design and methods

### Subjects

Included in this study were 70 pregnant women with confirmed GDM and 70 normal pregnant women without GDM as control at 24–28 weeks. All controls were matched (1:1) to GDM women by age groups: 24–27 years (*n* = 13), 27–30 years (*n* = 25), 30–33 years (*n* = 18), and 33–35 years (*n* = 14).

The diagnosis of GDM was according to the American Diabetes Association criteria with a 75-g oral glucose tolerance test at 24–28 weeks of pregnancy, with the cutoff value being >5.1 mmol/L at fasting, >10.0 mmol/L at 1 h, and >8.5 mmol/L at 2 h^12^.

The inclusion criteria were: (1) single pregnancies and (2) fasting plasma glucose <5.6 mmol/L before pregnancy. The exclusion criteria were: (1) pregnant women with previously known medical complications during pregnancy such as DM 1 or 2, hyperthyreosis, hypothyroidism, polycystic ovary syndrome, and inflammatory diseases; (2) multiple pregnancy; and (3) women who were treated with hormones or drugs that may affect glucose concentrations.

The study protocol was approved by the local ethics committee, and informed consent was obtained from all participants.

Some clinical features including the gestational age at delivery, maternal age, pre-gestational weight, height, and pre-body mass index (BMI) were included for analysis and comparison.

Blood glucose level was determined by using a Roche automatic biochemical analyzer (Roche Diagnostics, Mannheim, Germany). Malondialdehyde (MDA), superoxide dismutase (SOD), and glutathione (GSH) were used as markers of OS injury. Anti-OS enzymes SOD, GSH, and MDA levels were detected according to the instruction of the kits (Jiancheng Bioengineering Institute, Nanjing, China). The results are expressed as μmol/L, U/mL, and mg/L.

Electrochemical luminescence immunoassay (Roche Diagnostics, Mannheim, Germany) was used to test fasting insulin and cortisol levels. Plasma epinephrine (E), noradrenaline (NE), glucagon, and cortisol levels were determined by radioimmunoassay (Roche Diagnostics, Mannheim, Germany). IR was assessed by homeostatic model assessment of IR (HOMA-IR) formula, and HOMA-IR was used to evaluate IR in pregnant women^13^.

Except for 1- and 2-h blood glucose, all blood samples were taken at 24–28 weeks of gestation and fasting for more than 8–10 h. Blood samples were collected from the veins of the upper limbs. Centrifuged blood and retained supernatant. All indexes are serological indexes.

### Statistical analysis

Continuous variables of normal distribution data are presented as mean ± standard error. IQR was usesd to describe continuous variables of non-normal distribution. Student’s *t* test for independent samples was used to identify between-group differences. Data that were not normally distributed were log transformed before analysis. Pearson’s correlation coefficient after log transformation was used to compare OS injury, stress hormones, and HOMA-IR between the two groups. *P* <0.05 was considered statistically significant. Statistical analysis was done using SPSS 16.0 (SPSS Inc., Chicago, IL, USA).

## Results

### Maternal characteristics of Control and GDM women

The maternal characteristics including the gestational age and maternal age at delivery were similar in Control and GDM groups at entry to the study. Pre-gestational BMI in the GDM group was 7.04% higher than that in the Control group (*p* < 0.05) (Table 1).

**Table 1:** Maternal characteristics in Control and GDM women.

	Control	GDM
Gestational age at screening (weeks)	26.42 ± 2.23	27.76 ± 1.96
Maternal age (years)	28.43 ± 2.64	29.18 ± 2.25
Pre-gestational BMI (kg/m^2^)	24.81 ± 6.02	26.15 ± 6.83^a^

*BMI* body mass index.

Values are expressed as mean ± standard error.

^a^*P* < 0.05 vs. Control group.

### Plasma glucagon, E, NE, and cortisol concentrations in Control and GDM women

The content of glucagon, E, and NE in the GDM group was increased by 22.42%, 36.82%, and 35.09% respectively, as compared with that in the Control group (*p* <0.05, <0.01, <0.01). The same trend was observed in cortisol. Compared with the Control group, the content of cortisol in the GDM group was increased by 8.21%, showing no significant difference between the two groups (*p* > 0.05) (Table 2).

**Table 2:** Stress hormones, oxidative stress, and HOMA-IR injury in Control and GDM women.

	Control	GDM
Glucagon (mU/L)	140.52 ± 22.13	172.05 ± 20.61b
E (ng/L)	348.56 ± 99.56	476.89 ± 111.09^a^
NE (ng/L)	148.57 ± 51.13	200.70 ± 50.08^a^
Cortisol (nmol/L)	464.62 ± 110	502.75 ± 124
MDA (μmol/L)	11.02 ± 3.42	16.86 ± 4.35^a^
SOD (U/mL)	147.65 ± 18.24	96.72 ± 16.27^b^
GSH (mg/L)	23.45 ± 6.05	19.28 ± 4.75^b^
HOMA-IR	1.96 ± 0.45	2.33 ± 0.31^b^

*E* epinephrine, *NE* noradrenaline, *MDA* malondialdehyde, *SOD* superoxide dismutase, *GSH* glutathione.

Values are expressed as mean ± standard error.

^a^*P* < 0.05 vs. Control group.

^b^*P* < 0.01 vs. Control group.

### OS injury in Control and GDM women

Compared with the Control group, the MDA content in the GDM group was increased by 52.99% (*p* < 0.01). Contrary to MDA, anti-oxidative enzymes SOD and GSH in the GDM group were decreased by 52.65% and 21.63%, respectively (*p* < 0.01), indicating that OS injury in the GDM group was more severe than that in the Control group (Table 2). Compared with the Control group, HOMA-IR index in the GDM group was increased by 27.32% (*p* < 0.01) (Table 2).

### Correlation of stress hormones and OS with HOMA-IR in Control and GDM women

In both groups, HOMA-IR was positively correlated with glucagon, E, NE, and cortisol (*p* < 0.05). MDA showed a significant positive correlation and SOD showed a negative correlation with HOMA-IR in both groups (both *p* < 0.05). However, no significant correlation between GSH and HOMA-IR was observed in both groups (*p* > 0.05) (Table 3).

**Table 3:** Correlation of stress hormones and oxidative stress with HOMA-IR.

	Glucagon		E		NE		Cortisol		MDA		SOD		GSH	
	*r*	*P*	*r*	*P*	*r*	*P*	*r*	*P*	*r*	*P*	*r*	*P*	*r*	*P*
GDM	0.11	0.04	0.27	0.03	0.42	0.01	0.28	0.02	0.35	0.04	−0.29	0.03	−0.18	0.23
Control	0.21	0.02	0.29	0.04	0.42	0.07	0.31	0.01	0.59	0.04	−0.48	0.01	−0.04	0.18

*E* epinephrine, *NE* noradrenaline, *MDA* malondialdehyde, *SOD* superoxide dismutase, *GSH* glutathione.

## Discussion

Our results suggest that stress hormones were strongly related to IR, which might be one of the important reasons for GDM. Pregnant women are usually in a stressful condition and more oxygen and energy are required during pregnancy, both of which may increase OS injury^14^. It was found in our study that OS injury in GDM women was more severe than that in Control women.

Serum MDA, SOD, and GSH levels suggest that OS injury in GDM women were more serious, which is consistent with the study of Shaw et al.^15^ and Amin et al.^16^ Other than OS, could stress hormone change during pregnancy be another reason for IR?

Knowing that E, NE, and cortisol are biological markers for stress^17,18^, and that glucagon also undergoes change in a stressful condition, we detected these stress-related hormones, and found that E, NE, and glucagon were significantly increased in GDM women, which may explain the stressful condition in GDM.

Stress hormones are also known as hyperglycemia hormones that can aggravate IR and increase blood glucose^19,20^. Prolonged serious OS injury could also induce IR and impair glucose metabolism. A previous study has demonstrated that enhanced OS injury may change stress habituation and increase E, NE, and cortisol levels^21,22^.

Cortisol is one of the important stress hormones. Cortisol could increase hepatic glucose production, aggravate β cell function, and decrease insulin secretion, all of which could lead to hyperglycemia^23,24^.

It was found in our study that the mean cortisol level in GDM women was insignificantly higher than that in Control, which is consistent with the finding of Da Costa et al.^25^. However, we found that cortisol was positively correlated with IR. In summary, OS injury aggravated IR in GDM women. In addition, stress hormone change in GDM women could reduce insulin secretion and was also positively correlated with IR, both of which lead to significant and persistent hyperglycemia in GDM women.

Other than stress hormones, some adipose tissue-derived hormones are also related to GDM^26^. Stress is a matter of debate. Stress is generally defined as the non-specific response of the body to any demand made upon it^27–29^. Stress could activate the hypothalamic-pituitary-adrenal (HPA) axis, induce cytological effects, and lead to diseases^30^.

Pregnancy is a process of slight and chronic stress. Compared with Control women, stress hormones were significantly increased in GDM women, and increasing hormones indicate that process of stress adaptation has delayed and wrecked. OS injury is one of the reasons for IR, leading to hyperglycemia. Other than OS injury, stress adaptation disturbance may be another mechanism to explain hyperglycemia in GDM women, which may be important to ensure a healthy pregnancy, and particularly beneficial for the prevention of pathologic glycemia-related diseases.

At present, the methods of evaluating IR, such as HOMA-IR and QUICKI, are widely used. They are all related to fasting insulin and fasting blood glucose. When evaluating IR in patients, our study uses HOMA-IR index, which has certain limitations. We measured the fasting insulin, fasting blood glucose, including insulin antagonistic hormones, glucagon, epinephrine, norepinephrine, cortisol, and so on. The changes of HOMA-IR and these antagonistic hormone indicators reflect the changes of IR in GDM patients. In the follow-up study, we will pay attention to the progress of IR evaluation.
